# 16 Weeks of Progressive Barefoot Running Training Changes Impact Force and Muscle Activation in Habitual Shod Runners

**DOI:** 10.1371/journal.pone.0167234

**Published:** 2016-12-01

**Authors:** Ana Paula da Silva Azevedo, Bruno Mezêncio, Alberto Carlos Amadio, Julio Cerca Serrão

**Affiliations:** Laboratory of Biomechanics, School of Physical Education and Sport, University of São Paulo, São Paulo, São Paulo, Brazil; University of Alabama at Birmingham, UNITED STATES

## Abstract

Short-term effects of barefoot and simulated barefoot running have been widely discussed in recent years. Consequences of adopting barefoot running for a long period, including as a training approach, still remain unknown. The present study evaluated the influence of 16 weeks of progressive barefoot running training on impact force and muscle activation in habitual shod runners. Six habitual shod runners (3 men and 3 women, 29.5 ± 7.3 years) were tested barefoot (BF) and shod (SH), before and after 16 weeks of progressive barefoot running training. Tests consisted of running on instrumented treadmill at 9 km/h, for 10 minutes in each experimental condition. Nine data acquisitions (10 s) of vertical ground reaction force (VGRF) and electromyographic (EMG) signal were conducted in each experimental condition for each test. BF training was effective to alter VGRF and EMG parameters of running in habitual shod runners, regardless of footwear condition (SH or BF). The magnitude of first peak of VGRF (Fy1) and the impulse of the first 50 ms decreased after training for BF and SH (p<0.01). The activation reduced from PRE to POST training for four muscles in BF running (p<0.001), whereas only muscle gastrocnemius lateralis decreased significantly its activation (p<0.01) in SH running. A 16-week progressive barefoot running training seems to be an effective training strategy to reduce impact force, improve shock attenuation and to decrease muscle activation intensity, not only in BF running, but also in SH running, although BF condition seems to be more influenced by BF training.

## Introduction

Research interest and participation in barefoot (BF) running has increased remarkably in recent years [1–4]. Many reasons seem to drive people to BF running, however, this popularity is mainly based on the belief that BF alters biomechanical parameters of running, improving impact forces attenuation, increasing performance and reducing injury risk [2,3,5–8].

In short-term, the effects of BF running on biomechanical parameters have been previously described. Changes in spatiotemporal variables [7,9–11], foot strike pattern [6,12–14] and joint kinematics [13,15–18] have been reported for BF running in both habitual shod and barefoot runners. In runners without experience in BF, impact forces seem to be increased during barefoot locomotion, suggesting increased risk of injuries compared to shod (SH) condition [5,9,19–22]. Probably as consequence of this increased external load, literature also reports greater amplitudes of muscle pre-activation [17], increased muscle activation [17,18,23] and altered muscle coordination in BF running [17,18,23]. Such data could mean that the absence of footwear could also represent a risk for runners and, additionally, a less efficient running economy [17,23], although the real influence of BF condition on running economy remains unclear.

Despite the potential benefits of BF running, literature lacks studies investigating the long-term effects of barefoot running. The few data available on literature about this issue are related to studies that investigated habitual SH runners under short periods of familiarization or running training programs based on simulated barefoot (through minimalist shoes) [24–28]. Evidence shows that 4–12 weeks of simulated barefoot running induced to reduced plantar pressure [26], changes in muscle activation [27], a mid/forefoot strike pattern [24,26] and improvements in running economy [29]. Although there have been studies on how people switch running form and shoes, as far as we know, no study investigated the long-term effects of BF running or the use of this strategy as training approach for habitual SH runners. Additionally, none of these studies investigated the chronic influence of BF running on impact forces and shock attenuation. Thus, the investigation of impact forces and lower limb muscles involved in running becomes crucial for the understanding of barefoot adaptation’s process in long-term, as well as of the use of this mechanical condition as training approach.

Shot-term studies in habitual BF runners suggest the human body could adapt to BF situation and get benefits from the chronic use of this way of locomotion [6,10,12,13,16,30]. Improved mechanical load control [6,10,12], reduced muscle activation and improved running economy [16,30] have been observed in habitual BF runners. Experienced BF runners presented improvements in shock attenuation, as smaller incidence of first peak of VGRF or reduced magnitude of impact peak of VGRF during unshod [6,10,12,13]. Additionally, experienced BF runners present alterations in muscles activation pattern and decreased activation intensity of some muscles, what could mean less energy cost [10,16,30]. Thereby, BF running arises as a possible training strategy to improve mechanical load control and muscle activation [6,31–33].

Therefore, the purpose of this study was to analyze the influence of 16 weeks of progressive BF running training on kinetics and activation of selected muscles of lower limbs in habitual SH runners. For this, parameters of vertical ground reaction force (VGRF) and electromyographic (EMG) signal obtained during BF and SH running will be compared before and after 16 weeks of progressive BF training. Improvements in shock attenuation and decreased muscle activation intensity are expected after training.

## Material and Methods

### Participants

This research was approved by the Ethics Committee of the School of Physical Education and Sport of the University of São Paulo (Protocol N° 17816613.9.0000.5391, approved on January 3^rd^ 2013) prior to recruitment of participants. Investigation was conducted according to the principles expressed in the *Declaration of Helsinki*. All participants read and signed an informed consent term. Experimental design was approved by the local ethics committee. The authors confirm that all ongoing and related trials for this intervention are registered (ClinicalTrials.gov Identifier: NCT02815826). The S1 and S2 Files present the Trend Checklist and the study protocol approved for this research.

**Fig 1:**
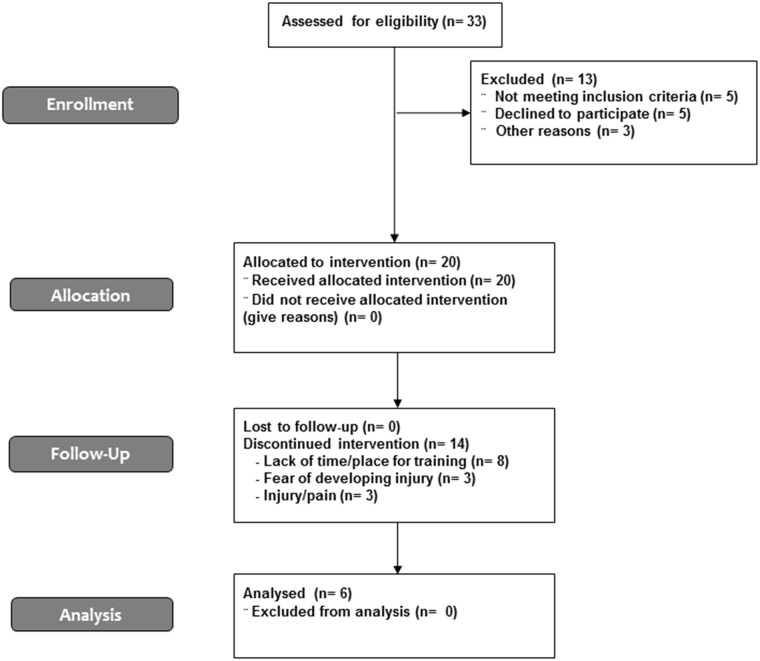
CONSORT flowchart of enrollment and follow-up.

This prospective study was performed from September 2012 to June 2013. After advertising the research runners’ communities, thirty three participants enrolled to the study but 13 were excluded (Fig 1). Twenty eligible participants (13 men and 7 women; 33.2 ± 6.4 years; 72.6 ± 14.2 kg; 1.72 ± 0.11 m) were recruited from a community of runners at the University of São Paulo, in Brazil. A questionnaire was used to collect information about running experience, average weekly running distance and previous lower limb injuries. Participants should be 18–40 years old, be experienced in running, but without experience in minimalist/barefoot running, had a minimum of 6 months of regular running training and a minimum of 6 months of experience in running on treadmills. Participants were not included if they had suffered any orthopedic injury in the last 12 months. Additionally, participants who presented habitual forefoot strike pattern, completed less than 80% of training and/or suffered any injury during training were excluded. Participants reported 5.6 years of experience in regular running training (0.5–22 years), weekly volume of 44.2 km (25–100 kilometers per week) and 4 training sessions per week (3–5 sessions per week).

### Intervention

According to literature [34–37], the transition from SH to BF running must be done through gradual changes of volume and intensity of stimulus. Thus, barefoot training was based on the weekly training volume (WTV) of each participant. The BF training volume and surfaces of training were controlled.

During the 16 weeks of training, participants kept their normal running training routine (wearing shoes), while they were introduced progressively to BF condition. Three training sessions were performed per week. Barefoot training started with 5% and ended with 20% of their WTV being performed without shoes (Table 1). Soft surfaces (i.e. sand and grass) were adopted in the beginning of training (week 1 to 8). From week 9 to 16, participants mixed soft with harder surfaces, including treadmill and asphalt, to accomplish the training. Training sessions were planned and prescribed by professionals, researches and participants together. All training sessions were supervised by the researchers.

**Table 1:** Barefoot running training progression (in % of weekly training volume—WTV).

Period (weeks)	Barefoot Training
**1**^**st**^ **to 4**^**th**^	Until 5%–walking in soft surfaces
**5**^**th**^ **to 8**^**th**^	5% to 10%–walking and light running (6–8 km/h) in soft surfaces
**9**^**th**^ **to 12**^**th**^	10% to 15%–light running (7–8 km/h) in mixed surfaces
**13**^**th**^ **to 16**^**th**^	15% to 20%–moderate running (8–10 km/h) in mixed surfaces

### Experimental protocol

Participants ran, before and after intervention, on a treadmill under two experimental conditions: barefoot and shod. Experimental condition order was randomized to avoid learning effects.

Each session test started with participants performing a maximum voluntary isometric contraction (MVIC) test for each muscle of interest [38,39]. The MVIC protocol consisted of 4 movement trials for each muscle: 2 submaximal trials of 10 seconds; 1 maximal trial of 5 seconds; and 1 maximal trial of 10 seconds. Then, a 5-minute period of warm-up at self-selected speed was performed on a treadmill. After that, participants ran (at 9km/h) during 10 minutes on an instrumented treadmill in both barefoot and shod conditions. Participants had a 2-minute interval between each trial while experimental condition was changed. The VGRF of both legs and EMG signal of tibialis anterior (TA), gastrocnemius lateralis (GL), long head of biceps femoris (BCF), rectus femoris (RF) and vastus lateralis (VL) of the right leg of each participant were obtained. These muscles were chosen due to their importance and contribution to running [40–42]. For shod trial, runners wore their own habitual running shoes. All shoes were in good conditions of use and had similar characteristics of construction.

Fig 1 presents the CONSORT flowchart of the study’s enrollment and follow-up.

### Equipment and data acquisition

The VGRF data was obtained by the Gaitway Instrumented Treadmill System (9810S1), composed by an instrumented treadmill with two piezoeletric platforms assembled on its surface (Trotter Treadmill Model 685, 01–06560201), an Analog/Digital (A/D) conversor (Keithley MetraByte DAS–1402) and the Gaitway Software (Versão 1.0x). The EMG signal was measured by the Lynx-EMG System 1000 (Lynx Electronic Technology LTDA.), composed by data acquisition EMG1000-VxRy module, an Analog/Digital (A/D) converter and the Lynx-AqDados program. Bipolar surface electrodes "Double" (Hal Industry and Trade LTDA), AgCl, were placed on muscle bellies and connected to active preamplifiers AX1010 (Lynx Electronic Technology LTDA.). Electrodes placement in each muscle occurred according to the criteria established by SENIAM (Surface Electromyography for the Non-Invasive Assessment of Muscles). Nine acquisitions (10 seconds each) of VGRF and EMG signal were recorded over the 10 minutes of test in each experimental condition, with sampling rate of 2600 Hz. An average of 20 steps (10 right and 10 left) were obtained in each trial acquisition.

### Signal processing and statistical analysis

The VGRF data was low pass filtered by a Butterworth filter (4th order, 90 Hz cutoff frequency). The start and end of each left and right step was determined using 30N threshold. VGRF was normalized by individual body weight, and time was normalized by total support time (0 to 100% of the support, 0.1% lag). The EMG signal was filtered by a digital Butterworth band pass filter of 4th order (cutoff frequency from 20 to 450Hz) and notch filters of 60Hz, 120Hz and 180Hz. After these procedures, RMS was calculated and data was normalized by the maximum voluntary isometric contraction (MVIC), obtained at the beginning of the test session, prior to the running test. The signal obtained between the 4^th^ and 8^th^ second of the last maximal trial of each muscle was used for normalization of EMG signal obtained during running. Examples of raw GRF and EMG data are available as Supporting Information (S3 File).

For VGRF analysis, the following variables were selected: magnitude of first peak of VGRF (Fy1); time to achieve first peak of VGRF (tFy1); loading rate (LR1), calculated by the ratio Fy1/tFy1; and impulse during the first 50 ms of stance (Imp50), calculated from the area under the curve GRF x Time, until 50 ms. Muscle activation intensity was assessed through calculation of the RMS (Root Mean Square) of EMG signal. This procedure was done for each muscle analyzed, during stance phase, for shod and barefoot running.

Data normal distribution was checked with the Kolmogorov-Smirnov test, while homoscedasticity was tested by Levene test. A two-way ANOVA for repeated measures was performed to compare shod and unshod running (condition), as well as pre and post intervention (moment). The Student-Newman-Keuls test was performed as post hoc test. The level of significance was set at 5%. The statistical analysis was performed with SigmaStat 3.5 (Systat Software Inc., USA). Statistical data reports are available as Supporting Information (S4 File).

## Results

### Participants

Of the 20 participants recruited for the study, only 6 runners (3 men and 3 women, 29.5 ± 7.3 years, 64.1 ± 11.0 kg, 1.68 ± 0.14 m) completed the study protocol and were included in the final analysis. Despite dropouts, the sample baseline characteristics remained similar. Dropouts from the study occurred due to: lack of time/place for training sessions (n = 8), fear of developing injury (n = 3) and injury/pain (n = 3), although one of these injuries was not related to BF training. Large samples are not common in researches involving a new, tough and long training program, as the present study. Although the reduced sample size, a sensitivity power analysis test was performed (alpha = 0.05; power = 0.8; number of measurements considered for each participant = 9; by G*Power v.3.1.9.2 free software, Dusseldorf, Germany) and a medium effect size (0.41) was observed [43–45].

### Ground reaction force

Significant interactions between condition (SH / BF) and moment (PRE / POST) were observed for all VGRF parameters, except for tFy1 (Table 2). Time to reach first peak (tFy1) presented only main effect of condition (p = 0.008), being 42.18% smaller for BF condition.

**Table 2:** Summary statistics (mean, standard deviation, F-value and p-value of interactions) of GRF data for shod (SH) and barefoot (BF) running, before (PRE) and after (POST) training.

*VARIABLES*	SH		BF		*F-value (interaction)*	*p-value (interaction)*
	PRE	POST	PRE	POST		
**Fy1 (BW)**	1.44 ± 0.06^a^	1.15 ± 0,06^a^ ^d^	1.63 ± 0.06^b^	0.89 ± 0.06^b^ ^d^	***12*.*616***	*0*.016*
**tFy1 (ms)**	34.10 ± 2.23	33.60 ± 2.23	20.30 ± 2.23	18.80 ± 2.23	*0*.*0579*	*0*.*819*^+^
**LR1 (BW/s)**	33.41 ± 2.36^c^	28.96 ± 2,36	62.65 ± 2.36^b^ ^c^	29.14 ± 2.36^b^	***37*.*816***	*0*.002*
**Imp50 (BW.ms)**	38.70 ± 0.98^a^	32.10 ± 0.98^a^	45.50 ± 0.98^b^	32.70 ± 0.98^b^	***9*.*801***	*0*.026*

*: significant interaction between shoe condition and moment.

^+^: significant main effect of moment.

^a^: difference between PRE and POST in SH running (post hoc).

^b^: difference between PRE and POST in BF running (post hoc).

^c^: difference between SH and BF at PRE moment (post hoc).

^d^: difference between SH and BF at POST moment (post hoc).

**Fig 2:**
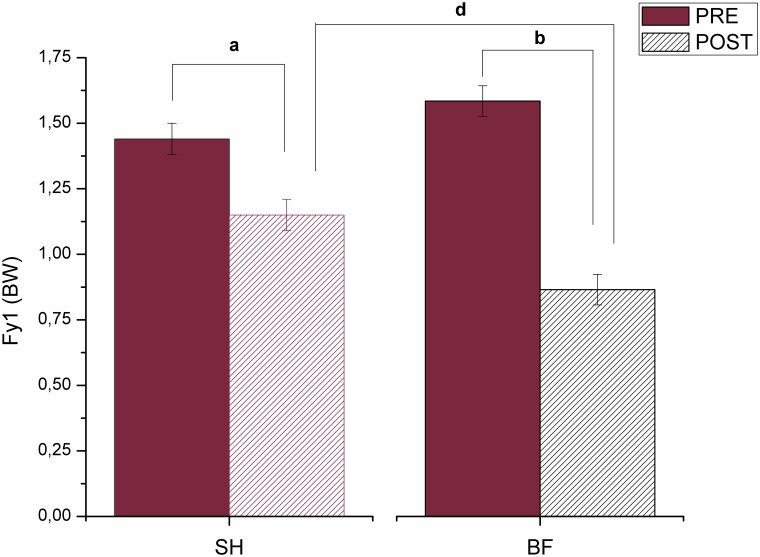
Mean and standard deviation values for the magnitude of first peak (Fy1) during running shod (SH) and barefoot (BF), in both PRE and POST training, where (^a^) means difference between PRE and POST in SH running; (^b^) means difference between PRE and POST in BF running; and (^d^) means difference between SH and BF at POST moment.

Post hoc test revealed differences between conditions and moments for variables analyzed. For Fy1, differences occurred between PRE and POST for both conditions of running (SH and BF) (p = 0.007 and p<0.001, respectively) and between SH POST and BF POST (p = 0.025) (Fig 2). In SH running, Fy1 decreased 20.1% from PRE to POST, while Fy1 presented a reduction of 45.4% from PRE in BF running. The Fy1 in BF POST was 22.6% smaller than SH POST.

**Fig 3:**
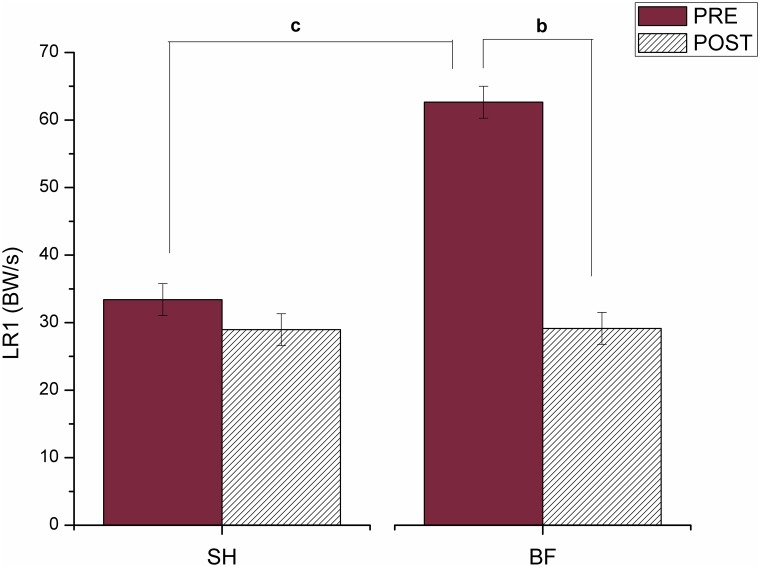
Mean and standard deviation values for the loading rate of first peak (LR) during running shod (SH) and barefoot (BF), in both PRE and POST training, where (^b^) means difference between PRE and POST in BF running; and (^d^) means difference between SH and BF at POST moment.

About LR (Fig 3), differences occurred between conditions before training (SH PRE and BF PRE) (p = 0.001) and between PRE and POST for BF running (p<0.001). Before training, LR in SH running was 46.7% smaller than in BF running. Additionally, LR reduced about 53.5% its value from PRE to POST in BF running.

**Fig 4:**
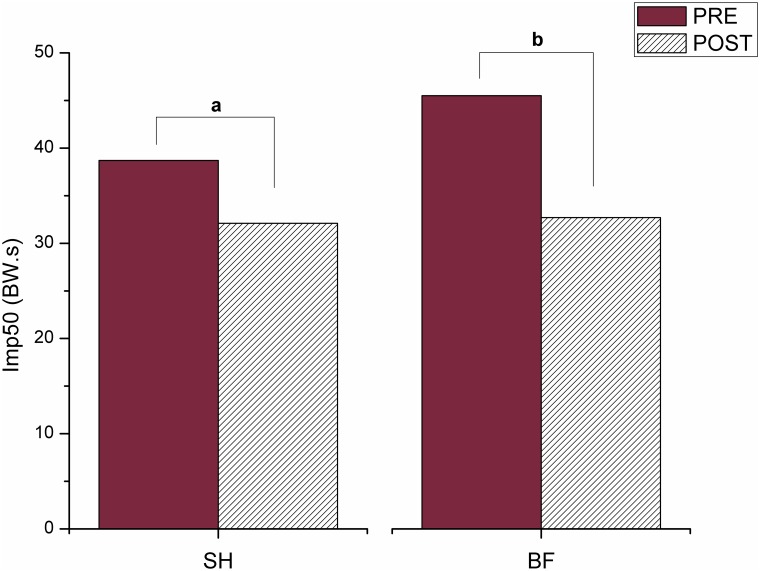
Mean and standard deviation values for the Impulse during the first 50 ms of stance (Imp50) during running shod (SH) and barefoot (BF), in both PRE and POST training, where (^a^) means difference between PRE and POST in SH running; and (^b^) means difference between PRE and POST in BF running.

Differences between PRE and POST in both conditions (p = 0.027 for SH and p<0.001 for BF) (Fig 4) were observed for Imp50. The Imp50 was 17% smaller after training in SH running. Similarly, Imp50 was 28.1% smaller after intervention in BF running. Additionally, a statistical trend of difference (p = 0.085) between SH and BF was observed before training. BF PRE was 17.57% higher than SH PRE. Probably, the post hoc test adopted in this study was not powerful enough to reveal statistical difference as sample size was reduced. This result is a reasonable explanation for the interaction observed for Imp50.

### Muscle activation

**Fig 5:**
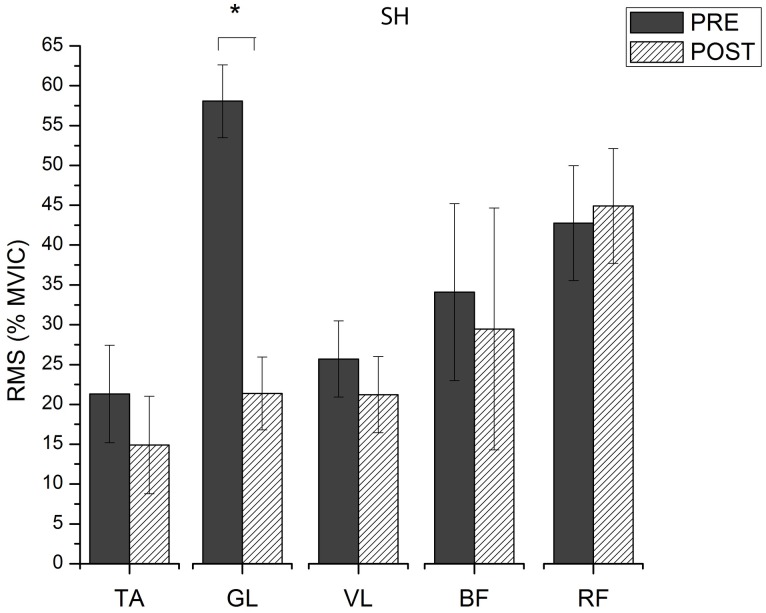
RMS values (% of MVIC) during stance phase of SH running, before (PRE) and after (POST) training.

**Fig 6:**
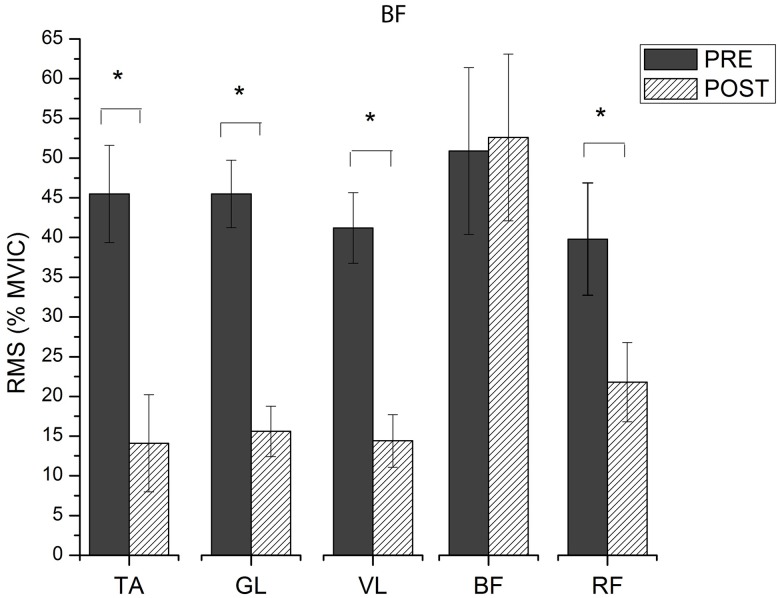
RMS values (% of MVIC) during stance phase of BF running, before (PRE) and after (POST) training.

**Fig 7:**
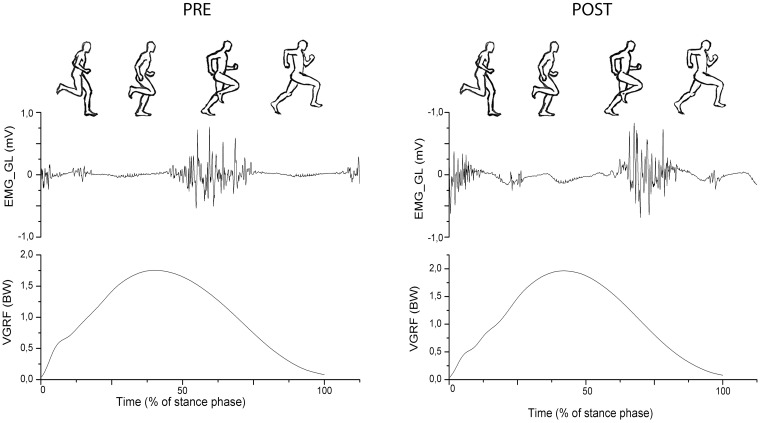
Illustrative average VGRF curves, raw EMG signal of m. gastrocnemius lateralis (GL) and running cycle of stance phase, for one participant, during BF running in before (PRE) and after (POST) training.

Significant interactions were observed for muscle activation. The muscle activation intensity decreased for most muscle as response to training, regardless footwear condition (p<0.01). Differences in RMS between the moments analyzed are presented on Figs 5 and 6. Only GL altered significantly its activation intensity during stance phase for SH running, decreasing 63% the RMS value from PRE to POST (Fig 5). All muscles, except BCF, reduced their activation intensity during stance phase of BF running after intervention (Fig 6). The TA decreased 69% the RMS from PRE to POST. Similarly, GL and VL showed a decrease of 66% and 65%, respectively, in their RMS when after BF training. A decrease of 45% in the RMS was also observed for RF in POST. The TA (111%), VL (131%) and BCF (115%) had greater values of RMS during stance phase in BF compared to SH running before training. After intervention, all muscles had similar activation intensity for both SH and BF, except BCF (157% greater RMS for BF then to SH). Fig 7 presents average VGRF curves and raw EMG signal obtained from GL during stance phase of one participant during BF running, before and after intervention, together with an illustrative sequence of running cycle during stance phase. S1 Table presents the summary statistics (mean, standard deviation and p-value of interactions) of RMS data.

## Discussion

This study aimed to investigate the effects of a 16-week progressive barefoot running training program on kinetics and EMG signal of lower limbs muscles in SH and BF running. Improved mechanical load control and decreased muscle activation intensity were expected after intervention, in both footwear conditions (SH and BF). The current investigation is, as far as we know, the first research to access the long-term progressive use of unshod running training.

The main finding of this study is that 16 weeks of progressive BF training induced changes to kinetic and EMG parameters of running regardless footwear condition, although the more substantial influence in muscle activation has occurred in BF condition. Another key finding of this research is that the human body was capable to adapt to unshod intervention. After training, similar or, even, lower impact force and muscle activation were observed for BF running compared to SH.

Results show that BF running is characterized by less efficient shock attenuation than SH condition in habitual shod runners, as described by previous research [9–11,13,22]. The higher value of LR for BF running before training suggests increased impact forces for this mechanical condition. Recent studies has shown that injured runners present higher values of LR [46,47]. Thus, since this VGRF variable is highly associated with some running injuries [46,48–51], BF running could characterize a initially harmful situation to habitual SH runners [1,9,20,35,36,49]. As expected, EMG signal followed the same behavior observed for external load. Corroborating to previous studies [10,17,23,52] our results showed that habitual SH runners presents higher muscle activation intensity under BF condition. Differences between BF and SH for RMS before training were marked in muscles associated with shock absorption, such as TA, VL and BCF [40,41,50]. This finding indicates the muscle behavior observed in this study was possibly a response to the greater impact forces presented by habitual SH runners during BF condition. In the presence of higher mechanical load, muscles may increase their activation to help in shock absorption. According to literature, greater muscle activation is related to injuries [51,53–56], high cost energy and less efficient running economy [10,17,23,25,30,57]. Therefore, habitual SH runners in their first attempt in this condition could have their protection and performance impaired.

The BF training induced to similar values of LR and Imp50 for BF and SH running, whereas BF condition presented smaller magnitude of impact peak of VGRF (Fy1) after training. Results show that runners adapted to BF condition potentially experiences diminished impact forces during BF running compared to SH running, corroborating to the findings reported by Divert et. al. [10], Lieberman et. al. [6] and Squadrone et. al. [12]. Accordingly, few differences were observed in muscle activation intensity between BF and SH running after intervention. Both ways of running presented similar RMS values after training for all muscles, except BCF. As such, runners adapted to the absence of footwear may be as efficient in BF as in SH running [57]. Additionally, BF running could be seen as a favorable training context to habitual BF runners, where they experiences the similar muscle activation of SH condition, but with decreased impact forces.

Both SH and BF running showed reduced values for variables related to shock attenuation, as Fy1 and Imp50, after training. These results suggest the unshod training improves mechanical load control and shock attenuation in BF and, also, in SH running. Considering these VGRF variables represent the impact forces and energy absorbed by human body structures, some studies associate them to running injuries [48–51]. Supported by these studies, results suggest an improved protection and reduced injury risk after BF training for both ways of running (BF and SH). Nevertheless, it is important to highlight the association between GRF variables and running injuries is still controversial and our assertion is a mere analysis of the potential of risk.

According to the literature, reduced impact forces are related to switching from rearfoot to a mid/forefoot strike pattern and to alterations in spatiotemporal parameters (as stride length and frequency) induced by BF condition [6,10,12,13,24,58]. As SH running also reduced impact forces after BF training, our results suggest these alterations induced by BF condition might be incorporated by runners during SH running. As expected, changes were observed for muscle activation in both SH and BF running as response to the 16-week progressive BF training. Almost all muscles reduced their activation intensity from PRE to POST training for both conditions. However, this reduction was statistically significant for 4 muscles in BF running, whereas only GL was significantly influenced by training in SH running. Although kinematic data was not measured, reduction in activation intensity of muscle may reflect the absence of impact shock to absorb induced by possible switching from rearfoot to a mid/forefoot strike pattern. Such results suggest neuromuscular adaptations in response to training, that could be related to more efficient muscle recruitment pattern and improved modulation of muscle activation in both passive and active phase of running [10,17,23,41,59,60]. As muscles play an important role as shock absorbers during running [40,41,50], the reduced RMS may reflect the neuromuscular response of Central Nervous System (CNS) to the diminished impact forces observed after training, mainly in BF running. Another possible reason for the reduced intensity of muscle activation after training may be an improvement in the stretch-shortening cycle. Researchers report the kinematics and foot strike pattern induced by unshod running possibly improves the use of storage elastic energy [6,16,57]. Results also suggest the chronic adaptations in muscle activation intensity to the 16 weeks of progressive unshod training seem to be more expressive in BF running. Due to movement specificity and learning effects (since all runners were habitual SH), BF running may have been more sensitive to our intervention than SH running. It is important to notice that many of these changes on GRF and EMG variables in habitual SH runners may be also observed for barefoot simulated running, achieved by minimalist shoes, but in different magnitudes [12,14,17,58,61,62]. The strike pattern is another relevant aspect that must be considered on the study of BF running training. Although strike pattern may limit BF effects [8,63], it seems to be also determined by footwear condition [64,65]. Due to its complexity, strike pattern appears as an issue that should be investigated more deeply.

Some limitations should be considered in interpretations of findings. First, the sample size that completed the study protocol may restrict interpretation of results. Indeed, the final sample size is small, but different scenery would not be possible with the experimental design adopted. The difficulty of maintaining participants involved in an experimental protocol based on arduous and long training, as barefoot running for 16 weeks, must be highlighted and considered. Notwithstanding, effect size was calculated to express the reliability and sensitivity of our results. The effect size is a complementary statistical tool usually adopted in order to reveal the size of effects [66], being particularly meaningful for our study to assure more reliability and certainty to our results, even with a small sample. Another limitation is that running tests were performed on treadmill, what could change running style and mechanical responses [41,51]. To minimize this limitation, runners experienced in treadmills were recruited and familiarization period was provided. The analysis of EMG signal, considering the entire stance phase, also appears as a limitation of the study. Nevertheless, our intention was to obtain information about the global effort exerted by muscles during running phase. The absence of a control group also appears as a limitation of the study. To reduce this limitation, all participants kept their normal running training routine, without significant change in training volume and intensity, in order to guarantee that the inclusion of BF intervention would be the only modification in their training periodization. Additionally, the mechanisms behind the changes observed in this study were not accessed. Further investigations about the influence of BF training on different biomechanical parameters, including kinematics and foot strike pattern, are encouraged. Finally, our results are protocol dependent and should be extrapolated to other situations carefully. Other populations and different BF interventions may induce distinct mechanical responses for the conditions tested.

Thus, this research provides information to better understand the adaptation’s process to BF condition and about the consequences of adopting BF running as training approach. A progressive BF training was effective to alter VGRF and EMG parameters of running in habitual shod runners, regardless footwear condition (SH or BF). Results suggest the use of BF condition could be an efficient training strategy to reduce impact forces and to decrease muscle activation intensity, not only in BF running, but also in SH running, although changes in muscle activation has been more expressive for BF condition. The BF condition arises as an option and practicable training approach to improve mechanical load control and to enhance muscle recruitment for both SH and BF running.

## Conclusion

A 16-week progressive barefoot (BF) training altered running kinetics and changed variables of ground reaction force (GRF) related to external forces in habitual shod runners. Additionally, muscle activation intensity of habitual shod runners was influenced by BF training. Alterations occurred in both shod (SH) and barefoot (BF) running. Hence, a progressive BF running training could be used as strategy to improve mechanical load control and shock attenuation in running, regardless footwear condition. Moreover, an intervention based on BF condition reduces muscle activity intensity in long-term, mainly in BF running, what could be a possible and useful approach to improve running economy.

## Supporting Information

S1 File Trend Statement Checklist of this paper. (PDF)

S2 File Study protocol submitted and approved by local ethic committee. (PDF)

S3 File Data used in the statistical analysis. (XLSX)

S4 File Statistical data reports from SigmaStat 3.5. (RTF)

S1 Table Summary statistics (mean and standard deviation) of RMS data (% MVIC) during stance phase for shod (SH) and barefoot (BF) running, before (PRE) and after (POST) training (p<0.05). (DOCX)
