# Correction: 16 Weeks of Progressive Barefoot Running Training Changes Impact Force and Muscle Activation in Habitual Shod Runners

**DOI:** 10.1371/journal.pone.0176426

**Published:** 2017-04-19

**Authors:** Ana Paula da Silva Azevedo, Bruno Mezêncio, Alberto Carlos Amadio, Julio Cerca Serrão

The Funding Statement appears incorrectly in the published article. The correct funding information is as follows: The University of São Paulo (USP) provided support for this research through the “USP in 2016 Olympic Games Program.” The authors also thank the Coordination of Improvement of People from Higher Education (Coordenação de Aperfeiçoamento de Pessoal de Nível Superior—CAPES) for financial support to publish this manuscript. The funders had no role in study design, data collection and analysis, decision to publish, or preparation of the manuscript.

## References

[1] Azevedo APdS, Mezêncio B, Amadio AC, Serrão JC (2016) 16 Weeks of Progressive Barefoot Running Training Changes Impact Force and Muscle Activation in Habitual Shod Runners. PLoS ONE 11(12): e0167234 doi:10.1371/journal.pone.0167234 27907069 10.1371/journal.pone.0167234 PMC5132300

