# Supplementary material for: 16 Weeks of Progressive Barefoot Running Training Changes Impact Force and Muscle Activation in Habitual Shod Runners
Source: PLoS One. 2016 Dec 1;11(12):e0167234. doi: 10.1371/journal.pone.0167234 (PMC5132300; doi:10.1371/journal.pone.0167234)
Supplement: S2 File — (PDF) [file pone.0167234.s002.pdf]

STUDY PROTOCOL

1 STUDY'S CHARACTERIZATION

This is a long-term study that aims to characterize unshod running through dynamic and electromyographic parameters, and to investigate the long-term effects barefoot adaptation. For this, a barefoot running training program will be implemented and evaluated for 4 month.

1.1 Participants

The following inclusion criteria will considered to participants' eligibility: Participants should be 18-40 years old, be experienced in running, but without experience in minimalist/barefoot running, had a minimum of 6 months of regular running training and a minimum of 6 months of experience in running on treadmills. Participants will not be included if they had suffered any orthopedic injury in the last 12 months. Additionally, participants who presented habitual forefoot strike pattern, completed less than 80% of training and/or suffered any injury during training were excluded.

Before the beginning of tests, all participants will be examined and answer an orthopedic anamnesis to ensure the integrity of human body structures. Additionally, a questionnaire will be used to collect information about running experience, average weekly running distance and previous lower limb injuries. All participants will informed about the research and will read and sign an informed consent term.

1.2 Intervention

Literature lacks of evidences about minimalist or barefoot running training programs. Therefore, the proposed intervention is based on few data found in literature and on suggestions provided by footwear manufacturers about how to transition from conventional to minimalist shoes. The purpose is to promote a safe transition to barefoot running.

According to literature (VIBRAM, 2012; CAUTHON; LANGER; CONIGLIONE, 2013; RIDGE et al., 2013), the transition from SH to BF running must be done through gradual changes of volume and intensity of stimulus. Thus, barefoot training was based on the weekly training volume (WTV) of each participant. The BF training volume and surfaces of training were controlled.

During the 16 weeks of training, participants kept their normal running training routine (wearing shoes), while they were introduced progressively to BF condition. Three training sessions were performed per week. Barefoot training started with 5% and ended with 20% of their WTV being performed without shoes. Soft surfaces (i.e. sand and grass) were adopted in the beginning of training (week 1 to 8). From week 9 to 16, participants mixed soft with harder surfaces, as treadmill and asphalt, to accomplish the training. Training sessions were prepared by professionals, researches and participants together. All training sessions were supervised by the researchers.

1.3 Equipment

To comply with the purposes of the study, a Biomechanical approach will be adopted. Dynamic and electromyographic parameters will be evaluated from running.

1.3.1 Dynamometry

The VGRF data was obtained by the Gaitway Instrumented Treadmill System (9810S1), composed by an instrumented treadmill with two piezoeletric platforms assembled on its surface (Trotter Treadmill Model 685, 01-06560201), an Analog/Digital (A/D) conversor (Keithley MetraByte DAS–1402) and the Gaitway Software (Versão 1.0x).

The Trotter treadmill has the following features and specifications: 138.9 cm x 49.5 cm canvas, speed variation from 0.8 to 20.0 km/h, inclination range from -5 to 20%, 3.0 HP motor (KISTLER, 1996).

The Gaitway System is composed of two piezoelectric force plates placed in serie under the canvas. This arrangement allows the acquisition of many steps in sequence and the discrimination each step according to the contact on the plates. There is device on treadmill that identifies right and left

steps through a retroreflective infrared signal and sends the data to the management software.
The A/D converter has the following features and specifications: 16-channel, 12-bit resolution, absolute accuracy of 0.01%, conversion time of 8,0μs maximum conversion time, typical value of conversion of 7,5μs, input impedance of >25M ohm, minimum value of common mode rejection of 100db for gain of 500, DMA gain 500 (0 to +20 mV for unipolar) and 500 (± 20 mV for bipolar).

1.3.2 Electromyography

The EMG signal was measured by the Lynx-EMG System 1000 (Lynx Electronic Technology LTDA.), composed by data acquisition EMG1000-VxRy module, an Analog/Digital (A/D) converter and the Lynx-AqDados program.

This equipment amplifies, filters and digitalizes until 28 channels of analog input. There are 24 channels for electromyographic signals (12 passive and 12 active) and 4 inputs for instrumentation. Each electromyographic channel has a differential amplifier (rejection common mode >100dB; Condition: sinusoidal signal 10Vpp, 60Hz) with a fixed gain of nominal value 1000, 10.000 MOhms of typical input impedance, a high-pass Butterworth filter of 1st order (attenuation below the cut frequency of 20dB/decade) with cutoff frequency of 1 Hz and a low pass filter of 2nd order Butterworth (attenuation above the cutoff frequency of 40dB / decade) with cutoff frequency at 1kHz.

The amplified and filtered signals are digitized by an A/D converter with 16-bit resolution and with programmable input range. Once digitalized, the signals pass through 1st order Butterworth high-pass and 2nd order Butterworth low-pass digital filters, selectable from 0.01 to 50 Hz and between 1000/500/300/200Hz, respectively. The sampling rate is programmable and identical for all channels, being 4.000 Hz the maximum possible value.

In this study, 5 active channels will be used, with the input range of the A/D converter programmed in +/-2V. System digitalizes data simultaneously with acquisition. After recorded, the EMG signals are digitally filtered by a high-pass Butterworth filter of 1st order and a low-pass Butterworth of 2nd order. The cutoff frequencies will be 20 and 500Hz, respectively. Notch filters will be

programmed to cut the frequency of the local power grid (60Hz) and its harmonics (120Hz and 180Hz).

Communication with the computer was done through network interface ETHERNET 10Mbits/s and supported by AqDados 7.02 software (Lynx Electronic Technology LTD.). This program allows setting name, units, input range and calibration of analog input channels.

Bipolar surface electrodes "Double" (Hal Industry and Trade LTDA), AgCl, will be placed on muscle bellies. This electrode is composed of circular electrodes of silver chloride (AgCl) mounted on an adhesive foam with a thickness of 2mm. The electrodes have 10mm diameter and 10mm distance between electrodes. The set has a little more than 12mm of solid electrolytic gel disc mounted under each electrode. This gel reduces the impedance between electrode and skin, and assists in the electrode attachment (it is adhesive).

The bipolar surface electrodes will be connected to active preamplifiers AX1010 (Lynx Electronic Technology LTDA.) with gain of 20 times. These preamplifiers have a balanced connector on one of their extremity that connects to the electromyography device, and two cables with claws on the other end, which connect to bipolar electrodes.

The muscles to be monitored will be: the tibialis anterior (TA), lateral gastrocnemius (GL), rectus femoris (RF), vastus lateralis (VL) and long head of biceps femoris (BF). These muscles were chosen because they describe the behavior of large muscle groups, which are responsible for control of the hip, knee and ankle during running phases. The monitoring of EMG signal will be carried out only for the right lower limb. Electrodes placement in each muscle will be done according to the criteria established by SENIAM (Surface Electromyography for the Non-Invasive Assessment of Muscles). Through pre-determined anatomical points, the appropriate place to attach the electrodes is identified.

Normalization of EMG data will be done through maximum voluntary isometric contraction (MVIC).

1.4 Synchronization procedures

To give a complete and adequate analysis of movement, the acquisition systems mentioned above will be used simultaneously. However, systems' synchronization will not occur directly.

To synchronize electromyographic and dynamic data, a synchronization unit located in Gaitway System will be used. EMG data recording will be manually initiated some seconds before the start of GRF data recording. However, when the recording of GRF data starts at Gaitway System, a 5V-signal will be send to the LYNX-EMG1000 electromyography. Then, such signal will be recorded along with the electromyographic signal. This allows identifying the beginning of GRF data record. The synchronization will be done in the data processing phase, when the EMG data of interest will be "cut" (based on the electrical pulse sent), and interpolated to the same sampling rate of Gaitway System. The acquisition frequency of 2000Hz will be adopted for both Lynx and Gaitway System.

1.5 Parameters

1.5.1 Dynamic

The variables selected to evaluate possible alterations induced by experimental conditions are related to the vertical component of Ground Reaction Force (VGRF).

The GRF is a tridimensional force received as response of the interaction between human body and ground. This force has the same magnitude of the force applied to the ground, but in opposite direction. VGRF (Fy) during running presents two peaks: the first peak (Fy1) relates to impact force received at contact with ground, providing information about the overload absorbed; the second peak (Fy2) relates to the propulsive phase of running, provides information about performance.

For VGRF analysis, the following variables were selected: magnitude of first peak of VGRF (Fy1); time to achieve first peak of VGRF (tFy1); and impulse during the first 50 ms of stance (Imp50), calculated from the area under the curve GRF x Time, until 50 ms.

1.5.2 Electromyographic

Muscle activation intensity was assessed through calculation of the RMS (Root Mean Square) of EMG signal. This procedure was done for each muscle analyzed, during stance and swing phase, for shod and barefoot running.

1.6 Experimental procedures

Participants will be evaluated before and after 4 months of barefoot running training. In each test session, participants will be evaluated running under two experimental conditions: shod and barefoot. The habitual conventional running shoe of each participant will be worn in shod condition. Data of shod and unshod running will be recorded in a single session. The order of conditions will be determined randomly.

Each test session will begin with preparation of participants: identification of sites to be placed electrodes in the muscles, trichotomy, skin scarification using sandpaper and skin cleaning through 96º Gl alcohol. Such procedures are required to remove sebum layer and the excess of dead skin cells, what decreases the impedance of electrode-skin interface and reduces the noise level in EMG signal. In sequence, surface electrodes will be placed on the skin of participants and fixed by adhesive tape in an attempt to prevent movements of equipment over the skin. For better adhesion, above the surface with the skin, a second fixation will be performed with another type of adhesive tape.

After surface electrodes attachment, participants will perform the MVIC test for each muscle selected. The exercises were: knee extension (m. vastus lateralis and rectus femoris), ankle dorsiflexion (m. tibialis anterior), ankle plantarflexion (m. gastrocnemius lateralis) and knee flexion (m. long head of biceps femoris). Based on protocols tested before (MCLEAN et al., 2003; BURDEN, 2010), the MVIC protocol consists of 4 movement trials for each exercise: 2 submaximal trials of 10 seconds; 1 maximal trial of 5 seconds; and 1 maximal trial of 10 seconds. All trials will be performed with verbal motivation. To normalize EMG signal obtained during running, the range from the 4th to 8th second of the last MVIC trial will be used (MCLEAN et al., 2003; BURDEN, 2010). The ground electrode will be placed at clavicle.

Finished the MVIC test, participants will start the running test on treadmill. For warm-up and adaptation to experimental conditions, running test will start with a 5-minute period of familiarization at self-selected speed. After familiarization, the first experimental condition will be evaluated through running for 10 minutes, at 9 k/h. GRF and EMG data will be recorded simultaneously at minutes 0, 5 and 10 (10 s each, at 2000 Hz).

After data acquisition for the first experimental condition (shod or barefoot), the procedures of treadmill test will be repeated in order to obtain biomechanical data during running under the other experimental condition.

1.7 Mathematical and statistical analysis of data

Signal processing will be performed at *Matlab 2009b* (Mathworks, USA). The VGRF data will be low pass filtered by a Butterworth filter (4th order, 90 Hz cutoff frequency). The start and end of each left and right step will be determined using 30N threshold. VGRF will be normalized by individual body weight, and time was normalized by total support time (0 to 100% of the support, 0.1% lag). The EMG signal will be filtered by a digital Butterworth band pass filter of 4th order (cutoff frequency from 20 to 450Hz) and notch filters of 60Hz, 120Hz and 180Hz. After these procedures, RMS will be calculated and data will be normalized by the maximum voluntary isometric contraction (MVIC), obtained at the beginning of the test session, prior to the running test. The signal obtained between the 4th and 8th second of the last maximal trial of each muscle will be used for normalization of EMG signal obtained during running.

Data normal distribution will be checked with the Kolmogorov-Smirnov test, while homoscedasticity will be tested by Levene test. A factorial analysis of variance for repeated measures will be performed to compare shod and unshod running, as well as pre and post intervention. The Tukey HSD test was performed as post hoc test. The level of significance will be set at 5%. Examination of first peak occurrence between experimental conditions will be done through Chi-Square test. The statistical analysis was performed with SAS 9.1 (SAS Institute Inc., USA).
