# Supplementary material for: 16 Weeks of Progressive Barefoot Running Training Changes Impact Force and Muscle Activation in Habitual Shod Runners
Source: PLoS One. 2016 Dec 1;11(12):e0167234. doi: 10.1371/journal.pone.0167234 (PMC5132300; doi:10.1371/journal.pone.0167234)
Supplement: S4 File — (RTF) [file pone.0167234.s004.rtf]

Notes and Legend:

Below, the statistical data report of each variable of GRF is presented, where:

-	“Condicao” means condition (SH and BF)
1 = SH
2 = BF

-	“Coleta” means moment (PRE and POST)
0 = PRE
	4 = POST


Fy1


Two Way Repeated Measures ANOVA (Two Factor Repetition)	

Data source: Data 1 in Notebook 1

General Linear Model

Dependent Variable: Col 6 

The following subjects were deleted from the calculations because of the pattern of missing data:
1.000	
3.000	
4.000	
5.000	
6.000	
7.000	
9.000	
11.000	
12.000	
13.000	
16.000	
17.000	
19.000	
20.000	


Normality Test:	Failed	(P < 0.050)

Equal Variance Test:	Passed	(P = 1.000)

Source of Variation	 DF 	 SS 	 MS 	  F 	  P 	
sujeito	5	0.957	0.191	7.148	0.152	
condicao	1	0.00678	0.00678	0.203	0.672	
condicao x sujeito	5	0.167	0.0334			
coleta	1	1.601	1.601	89.339	<0.001	
coleta x sujeito	5	0.0896	0.0179			
condicao x coleta	1	0.310	0.310	12.616	0.016	
Residual	5	0.123	0.0246			
Total	23	3.255	0.142			


Main effects cannot be properly interpreted if significant interaction is determined. This is because the size of a factor's effect depends upon the level of the other factor.

The effect of different levels of condicao depends on what level of coleta is present.  There is a statistically significant interaction between condicao and coleta.  (P = 0.016)

Power of performed test with alpha = 0.0500:  for condicao : 0.0500
Power of performed test with alpha = 0.0500:  for coleta : 1.000
Power of performed test with alpha = 0.0500:  for condicao x coleta : 0.783

Expected Mean Squares:
Approximate DF Residual for condicao = 5.000 
Approximate DF Residual for coleta = 5.000 
Approximate DF Residual for sujeito = 1.752 

Expected MS(condicao) = var(res) + 2.000 var(condicao x sujeito) + var(condicao)
Expected MS(coleta) = var(res) + 2.000 var(coleta x sujeito) + var(coleta)
Expected MS(sujeito) = var(res) + 2.000 var(condicao x sujeito) +2.000 var(coleta x sujeito) +4.000 var(sujeito)
Expected MS(condicao x sujeito) = var(res) + 2.000 var(condicao x sujeito)
Expected MS(condicao x coleta) = var(res) + var(condicao x coleta)
Expected MS(coleta x sujeito) = var(res) + 2.000 var(coleta x sujeito)
Expected MS(Residual) = var(res)

Least square means for condicao : 
Group	Mean	
1.000 	1.295	
2.000 	1.262	
Std Err of LS Mean = 0.0528

Least square means for coleta : 
Group	Mean	
0.000	 1.537	
4.000	 1.020	
Std Err of LS Mean = 0.0386

Least square means for condicao x coleta : 
Group	Mean	
1.000 x 0.000	 1.440	
1.000 x 4.000	 1.151	
2.000 x 0.000	 1.634	
2.000 x 4.000 	0.890	
Std Err of LS Mean = 0.0640


All Pairwise Multiple Comparison Procedures (Student-Newman-Keuls Method) :

Comparisons for factor: condicao
Comparison	Diff of Means	p	q	P	P<0.050	
1.000 vs. 2.000 	0.0336	2	0.636	0.672	No	


Comparisons for factor: coleta
Comparison	Diff of Means	p	q	P	P<0.050	
0.000 vs. 4.000 	0.517	2	13.367	<0.001	Yes	


Comparisons for factor: coleta within 1
Comparison	Diff of Means	p	q	P	P<0.05	
0.000 vs. 4.000	0.289	2	4.858	0.007	Yes	


Comparisons for factor: coleta within 2
Comparison	Diff of Means	p	q	P	P<0.05	
0.000 vs. 4.000	0.744	2	12.499	<0.001	Yes	


Comparisons for factor: condicao within 0
Comparison	Diff of Means	p	q	P	P<0.05	
2.000 vs. 1.000	0.194	2	2.787	0.078	No	


Comparisons for factor: condicao within 4
Comparison	Diff of Means	p	q	P	P<0.05	
1.000 vs. 2.000	0.261	2	3.753	0.025	Yes	


tFy1


Two Way Repeated Measures ANOVA (Two Factor Repetition)	

Data source: Data 1 in Notebook 1

General Linear Model

Dependent Variable: Col 7 

The following subjects were deleted from the calculations because of the pattern of missing data:
1.000	
3.000	
4.000	
5.000	
6.000	
7.000	
9.000	
11.000	
12.000	
13.000	
16.000	
17.000	
19.000	
20.000	


Normality Test:	Failed	(P < 0.050)

Equal Variance Test:	Passed	(P = 1.000)

Source of Variation	 DF 	 SS 	 MS 	  F 	  P 	
sujeito	5	0.000226	0.0000451	0.553	0.735	
condicao	1	0.00123	0.00123	18.039	0.008	
condicao x sujeito	5	0.000340	0.0000681			
coleta	1	0.00000646	0.00000646	0.149	0.716	
coleta x sujeito	5	0.000217	0.0000434			
condicao x coleta	1	0.00000173	0.00000173	0.0579	0.819	
Residual	5	0.000150	0.0000299			
Total	23	0.00217	0.0000943			


The difference in the mean values among the different levels of condicao is greater than would be expected by chance after allowing for effects of differences in coleta.  There is a statistically significant difference (P = 0.008).  To isolate which group(s) differ from the others use a multiple comparison procedure.

The difference in the mean values among the different levels of coleta is not great enough to exclude the possibility that the difference is just due to random sampling variability after allowing for the effects of differences in condicao.  There is not a statistically significant difference (P = 0.716).

The effect of different levels of condicao does not depend on what level of coleta is present.  There is not a statistically significant interaction between condicao and coleta.  (P = 0.819)

Power of performed test with alpha = 0.0500:  for condicao : 0.912
Power of performed test with alpha = 0.0500:  for coleta : 0.0500
Power of performed test with alpha = 0.0500:  for condicao x coleta : 0.0500

Expected Mean Squares:
Approximate DF Residual for condicao = 5.000 
Approximate DF Residual for coleta = 5.000 
Approximate DF Residual for sujeito = 4.485 

Expected MS(condicao) = var(res) + 2.000 var(condicao x sujeito) + var(condicao)
Expected MS(coleta) = var(res) + 2.000 var(coleta x sujeito) + var(coleta)
Expected MS(sujeito) = var(res) + 2.000 var(condicao x sujeito) +2.000 var(coleta x sujeito) +4.000 var(sujeito)
Expected MS(condicao x sujeito) = var(res) + 2.000 var(condicao x sujeito)
Expected MS(condicao x coleta) = var(res) + var(condicao x coleta)
Expected MS(coleta x sujeito) = var(res) + 2.000 var(coleta x sujeito)
Expected MS(Residual) = var(res)

Least square means for condicao : 
Group	Mean	
1.000 	0.0339	
2.000	 0.0196	
Std Err of LS Mean = 0.00238

Least square means for coleta : 
Group	Mean	
0.000	 0.0272	
4.000	 0.0262	
Std Err of LS Mean = 0.00190

Least square means for condicao x coleta : 
Group	Mean	
1.000 x 0.000	 0.0341	
1.000 x 4.000	 0.0336	
2.000 x 0.000 	0.0203	
2.000 x 4.000	 0.0188	
Std Err of LS Mean = 0.00223


All Pairwise Multiple Comparison Procedures (Student-Newman-Keuls Method) :

Comparisons for factor: condicao
Comparison	Diff of Means	p	q	P	P<0.050	
1.000 vs. 2.000 	0.0143	2	6.007	0.008	Yes	


Comparisons for factor: coleta
Comparison	Diff of Means	p	q	P	P<0.050	
0.000 vs. 4.000 	0.00104	2	0.545	0.716	No	


Comparisons for factor: coleta within 1
Comparison	Diff of Means	p	q	P	P<0.05	
0.000 vs. 4.000 	0.000500	2	0.202	0.889	No	


Comparisons for factor: coleta within 2
Comparison	Diff of Means	p	q	P	P<0.05	
0.000 vs. 4.000	 0.00158	2	0.637	0.662	No	


Comparisons for factor: condicao within 0
Comparison	Diff of Means	p	q	P	P<0.05	
1.000 vs. 2.000	 0.0138	2	4.818	0.008	Yes	


Comparisons for factor: condicao within 4
Comparison	Diff of Means	p	q	P	P<0.05	
1.000 vs. 2.000	 0.0148	2	5.194	0.006	Yes	


LR


Two Way Repeated Measures ANOVA (Two Factor Repetition)	

Data source: Data 1 in Notebook 1

General Linear Model

Dependent Variable: Col 8 

The following subjects were deleted from the calculations because of the pattern of missing data:
1.000	
3.000	
4.000	
5.000	
6.000	
7.000	
9.000	
11.000	
12.000	
13.000	
16.000	
17.000	
19.000	
20.000	


Normality Test:	Failed	(P < 0.050)

Equal Variance Test:	Passed	(P = 1.000)

Source of Variation	 DF 	 SS 	 MS 	  F 	  P 	
sujeito	5	2492.803	498.561	2.713	0.118	
condicao	1	1297.799	1297.799	10.125	0.024	
condicao x sujeito	5	640.884	128.177			
coleta	1	2161.225	2161.225	24.256	0.004	
coleta x sujeito	5	445.495	89.099			
condicao x coleta	1	1265.879	1265.879	37.816	0.002	
Residual	5	167.374	33.475			
Total	23	8471.459	368.324			


Main effects cannot be properly interpreted if significant interaction is determined. This is because the size of a factor's effect depends upon the level of the other factor.

The effect of different levels of condicao depends on what level of coleta is present.  There is a statistically significant interaction between condicao and coleta.  (P = 0.002)

Power of performed test with alpha = 0.0500:  for condicao : 0.682
Power of performed test with alpha = 0.0500:  for coleta : 0.971
Power of performed test with alpha = 0.0500:  for condicao x coleta : 0.998

Expected Mean Squares:
Approximate DF Residual for condicao = 5.000 
Approximate DF Residual for coleta = 5.000 
Approximate DF Residual for sujeito = 6.627 

Expected MS(condicao) = var(res) + 2.000 var(condicao x sujeito) + var(condicao)
Expected MS(coleta) = var(res) + 2.000 var(coleta x sujeito) + var(coleta)
Expected MS(sujeito) = var(res) + 2.000 var(condicao x sujeito) +2.000 var(coleta x sujeito) +4.000 var(sujeito)
Expected MS(condicao x sujeito) = var(res) + 2.000 var(condicao x sujeito)
Expected MS(condicao x coleta) = var(res) + var(condicao x coleta)
Expected MS(coleta x sujeito) = var(res) + 2.000 var(coleta x sujeito)
Expected MS(Residual) = var(res)

Least square means for condicao : 
Group	Mean	SEM	
1.000	 31.187	 3.268	
2.000 	45.895 	3.268	


Least square means for coleta : 
Group	Mean	
0.000 	48.031	
4.000 	29.051	
Std Err of LS Mean = 2.725

Least square means for condicao x coleta : 
Group	Mean	SEM	
1.000 x 0.000	 33.414	 2.362	
1.000 x 4.000	 28.960	 2.362	
2.000 x 0.000	 62.647	 2.362	
2.000 x 4.000	 29.142	 2.362	


All Pairwise Multiple Comparison Procedures (Student-Newman-Keuls Method) :

Comparisons for factor: condicao
Comparison	Diff of Means	p	q	P	P<0.050	
2.000 vs. 1.000 	14.707	2	4.500	0.025	Yes	


Comparisons for factor: coleta
Comparison	Diff of Means	p	q	P	P<0.050	
0.000 vs. 4.000	 18.979	2	6.965	0.005	Yes	


Comparisons for factor: coleta within 1
Comparison	Diff of Means	p	q	P	P<0.05	
0.000 vs. 4.000	4.454	2	1.394	0.352	No	


Comparisons for factor: coleta within 2
Comparison	Diff of Means	p	q	P	P<0.05	
0.000 vs. 4.000	 33.504	2	10.483	<0.001	Yes	


Comparisons for factor: condicao within 0
Comparison	Diff of Means	p	q	P	P<0.05	
2.000 vs. 1.000	 29.232	2	7.965	<0.001	Yes	


Comparisons for factor: condicao within 4
Comparison	Diff of Means	p	q	P	P<0.05	
2.000 vs. 1.000	0.182	2	0.0496	0.973	No	


Imp50


Two Way Repeated Measures ANOVA (Two Factor Repetition)

Data source: Data 1 in Notebook 1

General Linear Model

Dependent Variable: Col 4 

The following subjects were deleted from the calculations because of the pattern of missing data:
1.000	
3.000	
4.000	
5.000	
6.000	
7.000	
9.000	
11.000	
12.000	
13.000	
16.000	
17.000	
19.000	
20.000	


Normality Test:	Failed	(P < 0.050)

Equal Variance Test:	Passed	(P = 1.000)

Source of Variation	 DF 	 SS 	 MS 	  F 	  P 	
sujeito	5	0.000949	0.000190	2.340	0.141	
condicao	1	0.0000832	0.0000832	1.408	0.289	
condicao x sujeito	5	0.000295	0.0000591			
coleta	1	0.000563	0.000563	20.235	0.006	
coleta x sujeito	5	0.000139	0.0000278			
condicao x coleta	1	0.0000567	0.0000567	9.801	0.026	
Residual	5	0.0000289	0.00000578			
Total	23	0.00211	0.0000919			


Main effects cannot be properly interpreted if significant interaction is determined. This is because the size of a factor's effect depends upon the level of the other factor.

The effect of different levels of condicao depends on what level of coleta is present.  There is a statistically significant interaction between condicao and coleta.  (P = 0.026)

Power of performed test with alpha = 0.0500:  for condicao : 0.0804
Power of performed test with alpha = 0.0500:  for coleta : 0.940
Power of performed test with alpha = 0.0500:  for condicao x coleta : 0.666

Expected Mean Squares:
Approximate DF Residual for condicao = 5.000 
Approximate DF Residual for coleta = 5.000 
Approximate DF Residual for sujeito = 7.654 

Expected MS(condicao) = var(res) + 2.000 var(condicao x sujeito) + var(condicao)
Expected MS(coleta) = var(res) + 2.000 var(coleta x sujeito) + var(coleta)
Expected MS(sujeito) = var(res) + 2.000 var(condicao x sujeito) +2.000 var(coleta x sujeito) +4.000 var(sujeito)
Expected MS(condicao x sujeito) = var(res) + 2.000 var(condicao x sujeito)
Expected MS(condicao x coleta) = var(res) + var(condicao x coleta)
Expected MS(coleta x sujeito) = var(res) + 2.000 var(coleta x sujeito)
Expected MS(Residual) = var(res)

Least square means for condicao : 
Group	Mean	
1.000	 0.0354	
2.000	 0.0391	
Std Err of LS Mean = 0.00222

Least square means for coleta : 
Group	Mean	
0.000	 0.0421	
4.000	 0.0324	
Std Err of LS Mean = 0.00152

Least square means for condicao x coleta : 
Group	Mean	
1.000 x 0.000 	0.0387	
1.000 x 4.000	 0.0321	
2.000 x 0.000	 0.0455	
2.000 x 4.000	 0.0327	
Std Err of LS Mean = 0.000982


All Pairwise Multiple Comparison Procedures (Student-Newman-Keuls Method) :

Comparisons for factor: condicao
Comparison	Diff of Means	p	q	P	P<0.050	
2.000 vs. 1.000	 0.00372	2	1.678	0.289	No	


Comparisons for factor: coleta
Comparison	Diff of Means	p	q	P	P<0.050	
0.000 vs. 4.000 	0.00968	2	6.362	0.007	Yes	


Comparisons for factor: coleta within 1
Comparison	Diff of Means	p	q	P	P<0.05	
0.000 vs. 4.000	 0.00661	2	3.951	0.027	Yes	


Comparisons for factor: coleta within 2
Comparison	Diff of Means	p	q	P	P<0.05	
0.000 vs. 4.000	 0.0128	2	7.625	0.001	Yes	


Comparisons for factor: condicao within 0
Comparison	Diff of Means	p	q	P	P<0.05	
2.000 vs. 1.000	 0.00680	2	2.923	0.085	No	


Comparisons for factor: condicao within 4
Comparison	Diff of Means	p	q	P	P<0.05	
2.000 vs. 1.000	 0.000649	2	0.279	0.850	No	
