# Supplementary material for: 16 Weeks of Progressive Barefoot Running Training Changes Impact Force and Muscle Activation in Habitual Shod Runners
Source: PLoS One. 2016 Dec 1;11(12):e0167234. doi: 10.1371/journal.pone.0167234 (PMC5132300; doi:10.1371/journal.pone.0167234)
Supplement: S1 Table — (DOCX) [file pone.0167234.s005.docx]

**S4 Table.** Summary statistics (mean and standard deviation) of RMS data (% MVIC) during stance phase for shod (SH) and barefoot (BF) running before (PRE) and after (POST) training (p<0.05).

|  | **SH** | | **BF** | |  |
| --- | --- | --- | --- | --- | --- |
| *MUSCLES* | **PRE** | **POST** | **PRE** | **POST** |  |
| TA | 21,30 ± 6,12 ^c^ | 14,90 ± 6,12 | 45,50 ± 6,12 ^b c^ | 14,10 ± 6,12 ^b^ |  |
| GL | 40,20 ± 3,17 ^a^ | 14,80 ± 3,17 ^a^ | 45,50 ± 4,26 ^b^ | 15,60 ± 3,17 ^b^ |  |
| VL | 17,80 ± 3,32 ^c^ | 14,70 ± 3,32 | 41,20 ± 4,45 ^b c^ | 14,40 ± 3,32 ^b^ |  |
| BCF | 23,60 ± 7,69 ^c^ | 20,40 ± 10,50 ^d^ | 50,90 ± 10,50 ^c^ | 52,60 ± 10,50 ^d^ |  |
| RF | 29,60 ± 4,99 | 31,10 ± 4,99 | 39,80 ± 7,06 ^b^ | 21,80 ± 4,99 ^b^ |  |
